# Transcriptomics of Leaf Development in the Endangered Dioecious *Magnolia kwangsiensis*: Molecular Basis Underpinning Specialized Metabolism Genes

**DOI:** 10.3390/genes15030335

**Published:** 2024-03-04

**Authors:** Guole Qin, Xiaodong Li, Yingcan Qin, Linyuan Lu, Lixia Gao, Delong Guan

**Affiliations:** Guangxi Key Laboratory of Sericulture Ecology and Applied Intelligent Technology, School of Chemistry and Bioengineering, Hechi University, Hechi 546300, China; 06032@hcnu.edu.cn (G.Q.);

**Keywords:** Asian Magnoliaceae, *Magnolia kwangsiensis*, leaf development, comparative transcriptomics, polysaccharide biosynthesis

## Abstract

*Magnolia kwangsiensis*, a dioecious tree native to China, is recognized not only for its status as an at-risk species but also for its potential in therapeutic applications courtesy of its bioactive compounds. However, the genetic underpinnings of its leaf development and compound biosynthesis are not well documented. Our study aims to bridge this knowledge gap through comparative transcriptomics, analyzing gene expression through different leaf maturation stages. We studied the transcriptome of *M. kwangsiensis* leaves by applying RNA sequencing at juvenile, tender, and mature phases. We identified differentially expressed genes (DEGs) to explore transcriptional changes accompanying the developmental trajectory. Our analysis delineates the transcriptional landscape of over 20,000 genes with over 6000 DEGs highlighting significant transcriptional shifts throughout leaf maturation. Mature leaves demonstrated upregulation in pathways related to photosynthesis, cell wall formation, and polysaccharide production, affirming their structural integrity and specialized metabolic functions. Our GO and KEGG enrichment analyses underpin these findings. Furthermore, we unveiled coordinated gene activity correlating development with synthesizing therapeutically relevant polysaccharides. We identified four novel glycosyltransferases potentially pivotal in this synergistic mechanism. Our study uncovers the complementary evolutionary forces that concurrently sculpt structural and chemical defenses. These genetic mechanisms calibrate leaf tissue resilience and biochemical efficacy.

## 1. Introduction

The *Magnolia kwangsiensis*, colloquially known as the dioecious *magnolia* or by its other aliases, the finely stamened *Magnolia* and the Huan-Yong tree *Woonyoungia septentrionalis*, holds a singular place in the Magnoliaceae family as the sole plant recognized for its unisexual flowers and separate male and female trees [1,2]. This unique floral architecture has drawn considerable attention to the species from a botanical and a conservation perspective, necessitating in-depth exploration into its biology and ecological impact [1,3].

Endemic to China, the species marks its presence exclusively within the territories of Guangxi, Guizhou, and Yunnan [2,4]. Among these localities, the Huanjiang Mulun National Nature Reserve is distinguished for harboring one of the largest contiguous populations of dioecious Magnolias with a mere estimate of 200 mature individuals remaining—underscoring its rarity and the critical need for immediate protective measures [3,5]. According to the “Regulations on the Protection of Wild Plants”, which the People’s Republic of China issued, the dioecious magnolia is classified as a first-class national key protected wild plant. The “China Red Data Book of Endangered Species”, compiled by the Biodiversity Working Group of the China Council for International Cooperation on Environment and Development, further categorizes it as an endangered species. Globally, its vulnerability is recognized and echoed by the International Union for Conservation of Nature (IUCN), which lists the *M. kwangsiensis* as a critically endangered species within its universally accepted grading system of threatened plants [1,2,3,4]. These classifications underscore the urgency to sincerely preserve and understand this extraordinary species to ensure its continuity and well-being within the ecosystem.

Beyond its conservation imperative, the dioecious *magnolia* exhibits remarkable potential due to its ability to synthesize various biologically active compounds [6]. The evergreen nature of its leaves enhances its ornamental appeal, but more importantly, it allows for the production of plant essential oils and other derivatives [7,8]. These include compounds like flavonols and polysaccharides, which have been documented for their anti-inflammatory, antimicrobial, and antioxidant medicinal properties and hold therapeutic promise for various conditions, including beauty and skincare treatments, arthritis, and skin diseases [6,7,8].

Despite the significant historical and modern use of *M. kwangsiensis*, a comprehensive understanding of its genetics and the pathways by which it produces bioactive molecules remains elusive. Advances in biotechnology and the introduction of transcriptome analysis have catalyzed new research opportunities for species like *M. kwangsiensis*. Employing reference genome-guided transcriptomic analyses has emerged as a potent tool, providing deep insights into gene expression patterns and the complexities of biological functions [9,10]. Fortunately, the recent disclosure of the reference genome for *M. kwangsiensis* (Genbank ID: PRJNA1051600) has equipped researchers with an essential resource for delving into the molecular intricacies and exploring potential applications of this species.

Building upon this, our study undertook a comparative transcriptome analysis of *M. kwangsiensis* leaves. We systematically investigated differential gene expression and biosynthetic gene clusters across various developmental stages: juvenile buds, tender leaves, and mature leaves. By examining these variabilities, we aimed to elucidate the molecular mechanisms underlying the synthesis of unique bioactive compounds within the leaves of this plant. In doing so, we were able to provide a scientific basis for the conservation and sustainable utilization of *M. kwangsiensis*, paving the way for biotechnological advancements. This study aspires not only to offer new strategies for preserving this critically endangered species but also to expand our understanding of the dioecious magnolia’s distinctive genetic and metabolic characteristics.

## 2. Materials and Methods

### 2.1. Sample Collection

Leaf samples were collected from the Huanjiang Mulun National Nature Reserve, which is a biodiversity hotspot in China’s Guangxi region renowned for its conserved *M. kwangsiensis* population. The specimens for this study were meticulously gathered over three separate months—June, July, and September—to represent different phenological stages. For each collection trip, we visited the preserve and collected approximately 30 leaf samples from distinct trees distributed across the reserve to minimize environmental variation and ensure a representative sampling of the *M. kwangsiensis* population. Photos of the plant tissues used for RNA extraction are provided in Supplemental Figure S1.

### 2.2. RNA Extraction and Sequencing

Upon collection, the samples were immediately snap-frozen in liquid nitrogen to preserve RNA integrity and transported under cold conditions to the laboratory, where they were stored at −80 °C until RNA extraction. For RNA isolation, individual leaf samples for each group were processed separately. We used a Plant Total RNA Extraction Kit (Tiangen Biotech, Beijing, China), ensuring the protocol was meticulously followed to prevent RNA degradation and potential genomic DNA contamination. To eliminate residual genomic DNA contamination, the extracted RNA samples were treated with RNase-free DNase I (TaKaRa, Dalian, China) following the manufacturer’s protocol. The quality and quantity of the extracted RNA were assessed using the NanoDrop spectrophotometer and agarose gel electrophoresis. Sharp bands representing the 28S and 18S rRNA subunits were observed, with average intensity ratios of 2.1 ± 0.12 (mean ± SD, *n* = 3 biological replicates) across samples from different developmental stages and tissue sources. A pooled sample strategy was implemented for the transcriptome sequencing to minimize individual variation and gain a comprehensive view of the transcriptome. Equal quantities of RNA (~50 μg, from 5~10 leaf samples) from each group were pooled to create the composite samples for the respective groups. These pooled RNA samples were then utilized for library construction following the standard protocols of the sequencing facility.

### 2.3. Transcriptome Sequencing

The extracted RNA samples were fragmented into approximately 200–500 nucleotide fragments using divalent cations under high temperatures in the Illumina proprietary fragmentation buffer. First-strand cDNA was then synthesized from the fragmented RNA using SuperScript II reverse transcriptase (Invitrogen, Shanghai, China) and random primers, which was followed by second-strand cDNA synthesis with DNA polymerase I and RNase H. The double-stranded cDNA was purified with the QiaQuick PCR extraction kit (Qiagen, Venlo, The Netherlands). Illumina sequencing adapters were ligated to the cDNA fragments, which then underwent end-repair and the addition of a poly ‘A’ base followed by adapter ligation. The resulting cDNA libraries were amplified by PCR using Phusion DNA polymerase (NEB) and primers specific to the Illumina adapters. The insert size of the libraries was verified to be between 200 and 500 bp using an Agilent 2100 Bioanalyzer. These constructed cDNA libraries were pooled based on their concentrations. RNA sequencing of all three constructed libraries was performed by Shenzhen BGI Institute (BGI, Shenzhen, China) using the BGIseq500 platform. The output files were produced in FASTQ format, ensuring a baseline of quality and consistency in our sequencing data of approximately 3 GB. Each library was given an identifier code—KL2, 4, 5 corresponded to mature leaves, KL6, 7, 8 to buds, and KL9, 11 and 12 to tender leaves. Statistics of all sequencing were performed using the seqkit package.

### 2.4. Sequence Analysis and Bioinformatics

The sequence analysis followed the standard procedures for a reference-based transcriptome analysis. We employed a popular and efficient combination of software tools—HISAT2 for alignment, StringTie for transcript assembly, and EdgeR for differential expression analysis [11]. The sequence data were aligned to the *M. kwangsiensis* reference genome, which had been previously submitted and is available in public databases (bioproject: PRJNA1051600; biosample: SAMN38790308). The gene prediction file in the gtf format and the eggNOG-mapper annotation file were also adopted from the genome assembly (DOI:10.5281/zenodo.10259480, available at: https://zenodo.org/records/10259480, accessed on 23 December 2023).

For parameter settings, we adhered to the recommended guidelines provided by the developers of each software tool. HISAT2 was used to map RNA-seq reads to the reference genome with its default parameters, which were noted for their efficiency in handling plant genomes. StringTie was employed to reconstruct transcripts and estimate their abundances. Finally, EdgeR was selected to identify differential gene expression between groups with a stringent threshold set for differentially expressed genes (DEGs) as a log2 fold change (Log2FC) absolute value greater than 2 and an adjusted *p*-value less than 0.05.

Subsequent bioinformatics analysis, including sequence annotation, functional enrichment, and pathway analysis, were performed utilizing the OmicStudio tools (https://www.omicstudio.cn/tool, accessed on 22 December 2023) and custom scripts designed within our laboratory. The search for Arabidopsis homologous genes was performed using the reciprocal blast module in tbtools. The expression values of Arabidopsis stems, seeds, and leaves were downloaded from the Conekt database (https://conekt.sbs.ntu.edu.sg/, accessed on 23 December 2023) [12]. Gene Ontology (GO) terms were assigned to each transcript, providing insight into the samples’ underlying biological processes, cellular components, and molecular functions. KEGG databases were employed for pathway analysis to understand the complex biological interactions presented within our datasets [13]. The convenient TBtools v2.065 software was used to analyze functional enrichment and pathway analysis [12]. For data visualization and interpretation, we also used the Tbtools and OmicStudio tools (https://www.omicstudio.cn/tool, accessed on 23 December 2023) [14]. The integration of this platform allowed for adequate visualization of complex datasets, thereby facilitating the comprehension of the transcriptional landscape reflected in our results.

## 3. Results

### 3.1. Referenced Transcriptome Assembly

Nine magnolia leaf tissue samples representing three developmental stages (mature leaf samples KL2, KL4, and KL5; bud samples KL6, KL7, and KL8; and tender leaf samples KL9, KL11, and KL12) were used for sequencing. Approximately 3G 150 bp paired-end reads were generated for each sample. Over 93% of the raw reads from each sample were mapped back to the reference Magnolia genome with mapping rates ranging from 91.76% to 93.73% (Table 1). The high mapping rates across developmental stages indicated that the reference genome provided a suitable template for aligning reads. The transcriptomes of magnolia leaves at different developmental phases were thus comprehensively covered through RNA-Seq.

**Figure 1:**
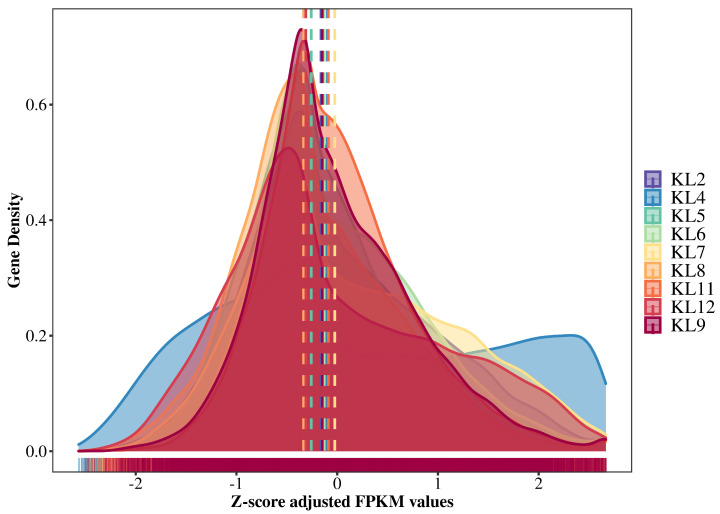
Density mapping of quantitative expression profiles across samples utilizing FPKM values. The horizontal axis of the map delineates the FPKM values that have been standardized through z-score adjustment, while the lateral axis corresponds to the gene density. Colored dashed lines within the figure denote the median FPKM values for the respective samples.

To assess the uniformity of sequencing results across samples and quantify gene expression, we calculated Fragments Per Kilobase of transcript per Million mapped reads (FPKM) values for each sample (Supplemental Table S1). Our results showed distinct but partially overlapping gene expression patterns among samples, as indicated by multiple non-identical peaks in the density distributions. Notably, the median FPKM values were remarkably consistent and closely aligned across samples, as evidenced by the proximity of lines in the density plots. Such consistent median FPKM values suggest high and reliable overall sample quality (Figure 1). The density plots indicated a high degree of similarity among the samples’ expressional profiles, reinforcing the suitability of our RNA-seq data for differential expression analysis. The closeness of the median FPKM values underscores that any variation among the samples is likely attributed to biological, rather than technical, factors. This level of expression uniformity is essential for ensuring confidence in the comparative analysis and provides a solid basis for further investigating the distinct transcriptional features and differences between the analyzed samples and groups.

### 3.2. Differential Gene Expression Analysis

**Figure 2:**
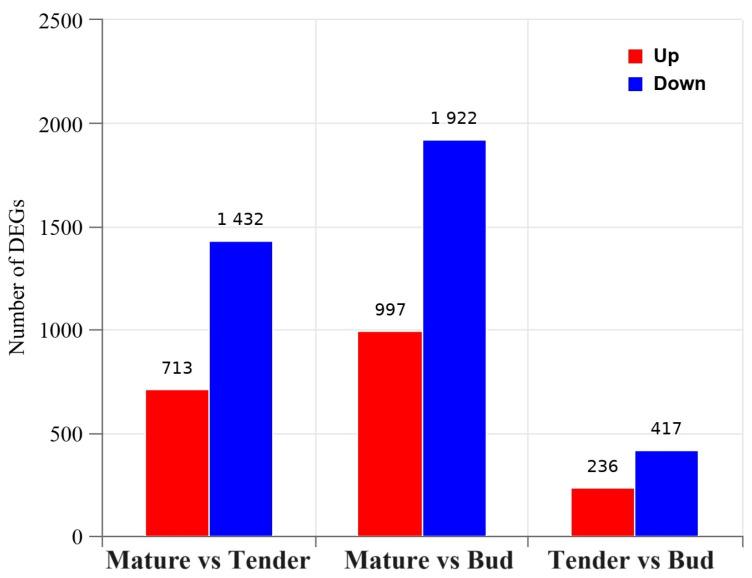
The number of differentially expressed genes among groups. The red and blue histograms represent the upregulated and downregulated genes, respectively.

To elucidate the molecular mechanisms underlying *M. kwangsiensis* leaf development and differentiation, a comprehensive analysis of differential gene expression between developmental stages was performed (Figure 2). Comparing tender leaves against buds revealed 653 differentially expressed genes (DEGs) with 236 upregulated and 417 downregulated. More DEGs were observed between mature and tender leaves with 2145 DEGs identified. Within this dataset, 713 genes showed elevated expression (upregulated), and 1432 genes showed lowered expression (downregulated) in mature versus tender leaves (Figure 2). The substantial number of DEGs highlights the intricate genetic reprogramming during maturation.

Moreover, a comparison of mature leaves versus buds detected the highest number of DEGs at 2919 (Figure 2). Among these, 997 genes were upregulated, indicating their potential roles in mature leaf advancement and functional establishment. Conversely, 1922 genes were expressed at lower levels (downregulated) in mature leaves compared to buds, suggesting the suppression or downscaling of specific pathways after the developmental phase encompassed by budding.

**Figure 3:**
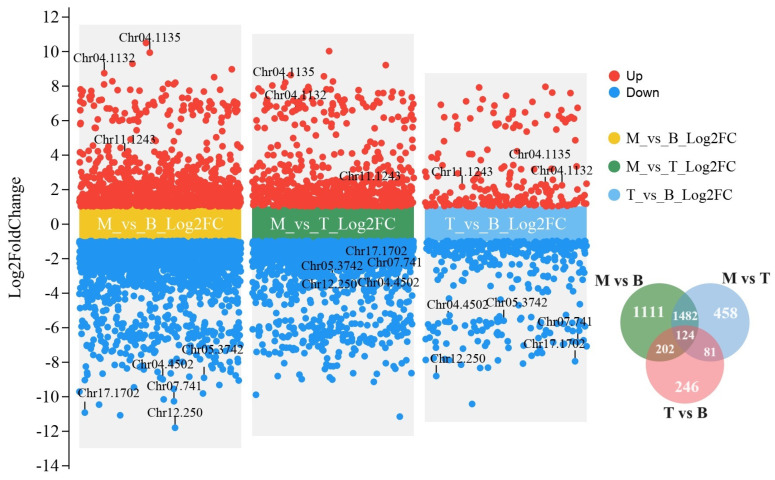
Volcano plots representing differentially expressed genes (DEGs) across three comparative groups in *M. kwangsiensis*. The groups are indicated as M (mature leaves), B (buds), and T (tender leaves). Red dots denote upregulated genes, while blue dots represent downregulated genes. The vertical axis reflects the log2 fold change (Log2FC) gene expression values. The Venn diagram classification of DEGs across the three groups is shown, highlighting the genes with the most significant variance common to all.

Then, a volcano plot was constructed better to visualize the extent and significance of gene expression changes. This plot was graphically represented for each comparative group—which included mature leaves, buds, and tender leaves—and revealed considerable variations in the fold changes of gene expression levels between these developmental stages. Our analysis indicated that within each group, the fold changes in FPKM values of genes exhibited a broad range. Notably, some genes displayed log2 fold changes (Log2FC) with absolute values surpassing 10, reflecting profound differences in gene expression intensity and potentially signaling substantial biological shifts during leaf development (Figure 3). The vast differences suggested by the observed Log2FC values provide compelling evidence for the existence of distinct regulatory mechanisms controlling the growth and development of *M. kwangsiensis* leaves.

Further delving into the data, 124 DEGs were extracted that were common across all three developmental stages (Figure 3). These shared DEGs are thought to play pivotal roles across the developmental spectrum and were subjected to closer scrutiny with the most significantly differentially expressed genes annotated in our subsequent analyses. Based on eggNOG sources, functional annotation and the GO enrichment of these DEGs revealed an array of gene functions (Supplemental Table S2). Genes were identified, like the ‘Origin recognition complex subunit’ (Chr01.89) involved in DNA replication initiation and genes belonging to the ‘synaptobrevin family’ (Chr01.171), which play roles in synaptic vesicle trafficking. Enrichment in histone genes, including multiple copies of ‘Histone H3’ (Chr01.179, Chr01.190), suggests modifications in the nucleosome structure and points toward active transcriptional regulation and DNA replication processes during all the stages in question.

Other significant genes included enzymes such as ‘phosphatase 2C’ (Chr01.752) and ‘7-deoxyloganetin glucosyltransferase-like’ (Chr02.417), which are vital for signal transduction and secondary metabolite biosynthesis, respectively. We also identified transcription factors like ‘protein breast cancer susceptibility 1 homolog’ (Chr01.4016), ‘High mobility group B protein’ (Chr02.1300), and ‘Transcription factor Myb_DNA-binding’ (Chr02.4189), which underline complex regulatory networks. Several components crucial for chromatin organization and dynamics were also found, such as ‘Dirigent proteins’ (Chr03.110, Chr03.111) central to plant secondary metabolism and other histone-related genes suggesting adjustments in chromatin accessibility and modification, which is often referred to as the ‘histone code’. The recognition of cytochrome P450 family members (Chr02.4745, Chr05.3742, Chr06.953) and kinases (Chr02.2959, Chr02.3594, Chr05.3242) points to cellular processes such as detoxification pathways and signal transduction being highly active during leaf development. Moreover, Tubulin genes (Chr05.3969, Chr08.2662) hint at cytoskeletal reorganization, which is crucial for cell division and elongation.

Identifying Arabidopsis orthologs for the Magnolia genes provides useful evolutionary context and enables expression comparisons during leaf development between the species. Among the differentiated transcriptome profiles from the *M. kwangsiensis* developmental stages, we searched for putative Arabidopsis orthologs. Overall, approximately 22,173 Arabidopsis genes exhibited Magnolia orthology, accounting for 61.7% of transcript sequences despite phylogenetic divergence between the taxa. Focusing on the differentially expressed upregulated cohorts comparing Mature versus Bud (M_vs_B), Mature versus Tender (M_vs_T), and Tender versus Bud (T_vs_B) stages, 831, 545, and 198 Magnolia orthologous Arabidopsis genes were identified, respectively. Strikingly, these ortholog subsets comprised 93.38% of M_vs_B, 76.43% of M_vs_T, and 83.89% of T_vs_B—indicating a broad representation of Arabidopsis leaf developmental genes among the Magnolia expression shifts.

**Figure 4:**
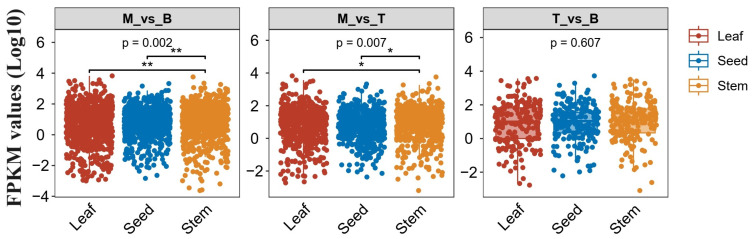
The expression levels of the *M. kwangsiensis* DEGs’ *A. thaliana* homologs across seeds, stems, and leaves in the *A. thaliana* transcriptome database. Genes marked with an asterisk (*) have significantly different expression levels among groups.

Probing the identified Arabidopsis orthologs in seed, stem, and leaf transcriptomic databases revealed consistent expression trends between the species with statistically robust differences (Figure 4). In M_vs_B and M_vs_T comparisons, Arabidopsis leaf expression proved markedly higher than stems (*p* < 0.01), which were in turn upregulated over seeds (*p* < 0.01). While T_vs_B followed the same pattern, differences lacked statistical power (Figure 4). Taken together, these results signify that Magnolia genes exhibiting pronounced expression shifts between leaf developmental states include ancient orthologous cohorts demonstrably active during Arabidopsis maturation—spotlighting core leaf development pathways conserved over vast evolutionary timescales between Magnoliids and Eudicots.

### 3.3. Functional Enrichment Analysis of DEGs

To understand the functional impact of differentially expressed genes linked to *M. kwangsiensis* leaf development, we conducted a comprehensive Gene Ontology (GO) enrichment analysis separately for up and downregulated genes (Supplementary Table S3). Our primary goal was identifying key biological processes strongly associated with mature leaf tissues. This allowed us to define the distinct transcriptional signature of mature foliage compared to earlier developmental phases.

**Figure 5:**
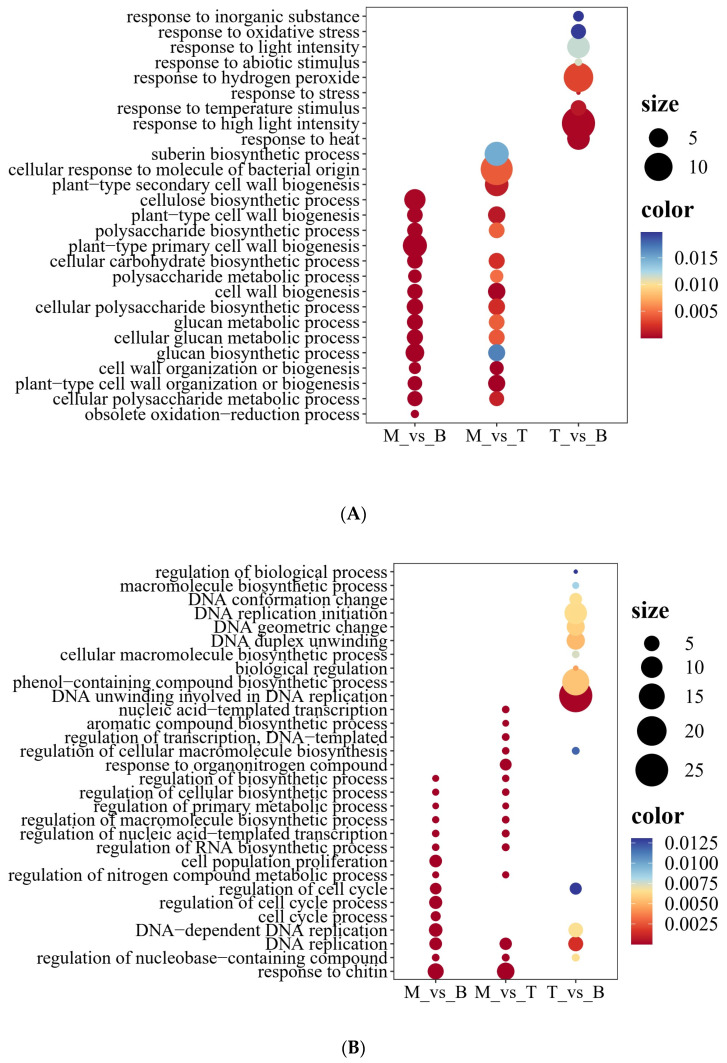
Gene Ontology (GO) functional enrichment analysis of upregulated and downregulated differentially expressed genes (DEGs) across three groups. The size of the points reflects the enrichment score, while the color indicates the *p*-value. (**A**) Enrichment analysis for upregulated genes. (**B**) Enrichment analysis for downregulated genes. The groups are indicated as M (mature leaves), B (buds), and T (tender leaves).

The upregulation patterns revealed an emphasis on carbohydrate and polysaccharide biosynthesis within mature leaves, marking a developmental shift toward generating biochemical precursors for structural reinforcement and bioactive molecule production (Figure 4A). These findings highlight the role of mature leaves in accumulating polysaccharides with therapeutic promise, anchoring future medical research into this ecologically and pharmacologically significant species. Compared to tender leaves and buds, mature leaves displayed an enriched expression of genes involved in ‘cellular polysaccharide biosynthesis’, including ‘cell wall biogenesis’, ‘plant-type cell wall organization’, and ‘glucan biosynthesis’. Such upregulation of ‘polysaccharide biosynthesis’ genes suggests an enhanced production of pharmacologically active polysaccharides in mature leaves (Figure 5A).

Further inspection showed an upregulation of ‘cell wall organization’ genes in mature leaves, indicating intensified cell wall strengthening and tissue restructuring during maturation, potentially enhancing defensive and structural integrity. In contrast, tender leaves predominantly upregulated stress response genes like ‘heat response’, ‘high light intensity response’, and ‘oxidative stress response’, marking a crucial adaptive phase for leaf acclimatization (Figure 5A). Unique to mature leaves versus buds was the upregulation of ‘cellulose biosynthesis’ genes integral to plant cell walls. Exclusive to mature versus tender leaves were terms like ‘secondary cell wall biosynthesis’ and ‘suberin biosynthesis’, indicating developmental shifts to strengthen structural scaffolds and establish internal leaf barriers (Figure 5A).

On examining the downregulation trends, we observed divergent patterns across the developmental spectrum. The GO analysis revealed the over-representation of processes such as response to chitin, cell cycle regulation, and DNA replication-related pathways among downregulated genes within mature leaves comparisons (Figure 5B). Mature leaves, in particular, underwent unique repression in cellular activities when compared to buds—which is a fact evident through the downregulation of genes linked to the regulation of nucleobase-containing compound metabolism and cell cycle processes. This suggests a pivot from active cell division within buds to specialization and functional differentiation within mature leaves. A broader downregulation of genes was observed in processes like the regulation of RNA biosynthetic process and nucleic acid-templated transcription within mature leaves. This aspect signifies a nuanced modulation of metabolic pathways ascending through the maturation stage (Figure 5B). An intriguing pattern emerged with the downregulation of genes involved in aromatic compound biosynthesis in mature leaves compared with tender leaves, indicating a potential decline in secondary metabolite production.

**Figure 6:**
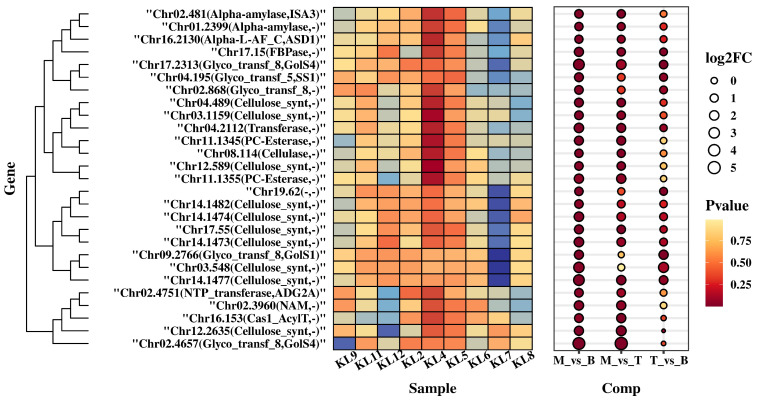
Gene expression profiles for 27 genes related to polysaccharides biosynthesis. The groups are indicated as M (Mature leaves), B (Buds), and T (Tender leaves). The heatmap on the left illustrates the relative expression levels of 27 genes associated with polysaccharide biosynthesis, as represented by FPKM values. The color intensity within each cell corresponds to the magnitude of the gene’s expression, with the scale indicating lower (blue) to higher (red) expression levels. On the left, hierarchical clustering is displayed based on z-score normalized FPKM values and utilizes Euclidean distance to groups. The bubble plot on the right side represents the Log2FC values. The size of each bubble indicates the magnitude of Log2FC, while the color denotes the statistical significance with the continuum ranging from non-significant (lighter) to highly significant *p*-values (darker).

Our analysis reveals the biosynthesis of pharmacologically significant compounds like flavonoids and polysaccharides. Based on our GO enrichment analysis, we cataloged the expression levels of 27 essential polysaccharide biosynthesis genes, revealing patterns reflecting hypothetical roles within this biosynthetic landscape. The ensuing table encapsulates Log2FC gene expression changes when comparing mature to tender leaves and buds (Figure 6). These quantified differences provide insight into expression variations potentially underlying polysaccharide synthesis machinery in *M. kwangsiensis*. We observed diverse up and downregulation, signposting a dynamic flux in orchestrating polysaccharide genes through development. Substantially upregulated genes in mature leaves included glycosyltransferase and cellulose synthesis isoforms, possibly signaling escalated polysaccharide production with advancing maturation. Our contrasting expression profiles pave the way for investigating complex regulatory networks governing leaf development, specialized metabolite accrual, and gene regulation driving secondary metabolite synthesis and storage in *M. kwangsiensis*.

### 3.4. KEGG Enrichment and Metabolite Pathway Analysis of DEGs

**Figure 7:**
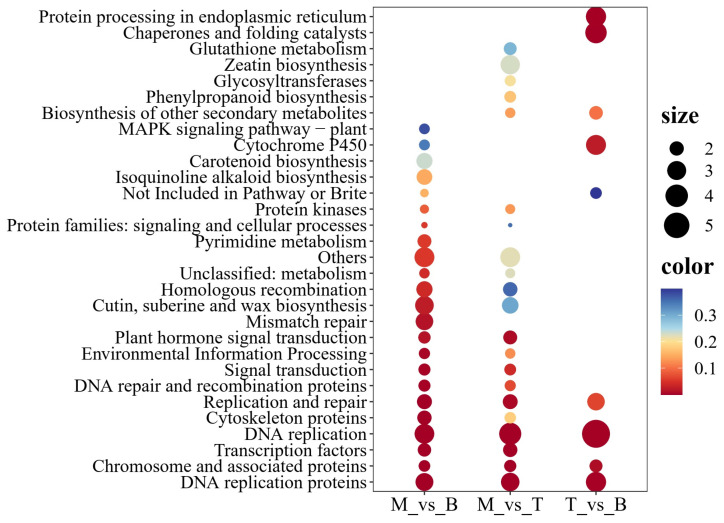
KEGG functional enrichment analysis of upregulated and downregulated DEGs across three groups. The size of the points reflects the enrichment score, while the color indicates the *p*-value. The groups are indicated as M (mature leaves), B (buds), and T (tender leaves).

From another angle, we look into the significant metabolite pathways implicated across the three pairwise comparisons to offer a snapshot of the complex regulatory and biosynthetic network modulating *M. kwangsiensis* leaf development. Therefore, a KEGG enrichment analysis was conducted, integrating upregulated and downregulated genes. Overall, enriched KEGG terms cross various significant biological functions and help elucidate the pathways dynamically engaged across different stages of leaf maturation. The balance between cellular proliferation, energy regulation, hormonal signaling, and secondary metabolite biosynthesis seems pivotal for properly executing leaf development and maturation processes and hints toward tailored metabolic priorities at each developmental stage (Figure 7).

In the comparison between mature leaves and buds, vital cellular processes such as ‘DNA replication proteins’, ‘DNA replication’, and ‘Mismatch repair’ were highlighted, indicating an active involvement of these pathways in the developmental transition. Notably, an enrichment in ‘Cutin, suberine, and wax biosynthesis’ indicated a modified epidermal barrier, which could be functional in mature leaf adaptation to environmental stresses. DNA replication pathways exhibited the highest enrichment for mature leaves versus tender leaves, followed by zeatin biosynthesis, which is integral to cytokinin production and plant growth regulation. Pathways related to ‘Signal transduction’, including ‘Plant hormone signal transduction’ and ‘MAPK signaling pathway—plant’ were also affected, emphasizing the importance of hormonal and stress signaling in leaf development (Figure 7).

In the contrast of tender leaves versus buds, the most significant enrichment was observed in the ‘DNA replication’ category, underscoring the high degree of cellular activity associated with leaf initiation and growth. As seen in the ‘Chaperones and folding catalysts’ grouping, chaperone-related functions are essential for protein folding and stability, indicating that protein maintenance and turnover play a critical role during early leaf development. ‘Protein processing in endoplasmic reticulum’ aligns with the need for post-translational modifications during these intense growth and differentiation phases. ‘Cytochrome P450’ enzymes known for their role in the biosynthesis of secondary metabolites were also considerably enriched, alluding to a diversity of biosynthetic activities in the budding phase (Figure 7).

While our primary aim revolved around the biosynthesis of bioactive compounds, no explicit polysaccharide biosynthesis pathway term directly emerged within the top enriched KEGG terms. However, glycosyltransferases were present in the mature versus tender leaf comparison, pointing toward enzymes, including polysaccharides, playing a crucial role in glycosidic compound synthesis. Glycosyltransferases transfer sugar moieties from activated donor molecules to specific acceptor molecules, forming glycosidic bonds—a reaction central to polysaccharide biosynthesis. This suggests a potential indirect association between glycosyltransferases and enhanced polysaccharide production in tender leaves compared to mature leaves.

**Figure 8:**
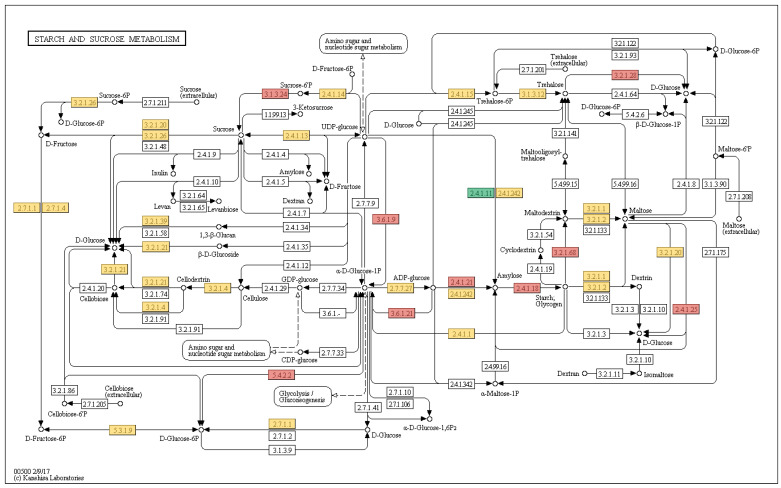
The diagram of the starch and sucrose metabolism pathway (KEGG map00500). Nodes with upregulated genes are highlighted in red, while those downregulated are marked in green. Nodes colored in yellow indicate the presence of both upregulated and downregulated genes.

To further investigate the mechanisms, the starch and sucrose metabolism pathway (map00500) was selected for closer inspection as a critical metabolic hub, with substantial enrichment of 161 differentially expressed genes, including multiple glycosyltransferases (Figure 8). These DEGs showed near-ubiquitous involvement across the pathway, participating in various stages and processes of starch and sucrose metabolism. Intriguingly, most nodes contained upregulated and downregulated genes, depicting complex, integrated regulation. Notably, critical nodes were associated with metabolites like amylose, cellulose, UDP-glucose and D-glucose—core components pertinent to synthesizing various bioactive metabolites. The expression alterations affecting these nodal points underlie a significant potential influence on biosynthesis, illustrating the dynamic interplay governing key synthetic processes. This pattern also suggests an association with polysaccharide synthesis in *M. kwangsiensis* leaves.

Moreover, the DEGs identified in the starch and sucrose metabolism pathway may be critical determinants of polysaccharide quality and quantity, influencing the medicinal properties of *M. kwangsiensis*. Integrating Gene Ontology (GO) enrichment and Kyoto Encyclopedia of Genes and Genomes (KEGG) pathway analysis allowed us to pinpoint critical genes contributing to the polysaccharide biosynthetic process, specifically those involved in starch and sucrose metabolism. Four genes were highlighted based on their functional annotations, each linking to pivotal roles within this biosynthetic pathway (Table 2).

The gene with ID Chr01.2399 is a prime example, which is annotated as encoding for the 1,4-α-glucan-branching enzyme. Although no specific gene name was provided, its Pfam domains, which include α-amylase, α-amylase_C, and CBM_48, confer the ability to interact with α-glucans. The 1,4-α-glucan-branching enzyme is responsible for introducing α-1,6-linked branches into the α-(1⟶4)-α-glucan polymer, which is crucial for the development and structuring of starch granules. The gene recorded as Chr02.481 is identified as Isoamylase 3 (*ISA3*), with Pfam domain profiles characteristic of the α-amylase family and CBM_48. Isoamylases are debranching enzymes that play a role in the biosynthesis and degradation of starch, making them essential for maintaining starch polysaccharides’ proper structure and functionality.

For gene Chr02.4751, a functional description highlights its significant role in starch synthesis. This gene encodes an enzyme, referred to as *ADG2A*, that catalyzes the formation of ADP-glucose. The ADP-glucose acts as an activated glycosyl donor in the biosynthesis of starch polysaccharides, utilizing Glc-1-P and ATP for the reaction, and the NTP_transferase Pfam domain suggests a mechanism that involves nucleoside triphosphate transferases. Moreover, the gene denoted as Chr04.195 falls within the glycosyltransferase 1 family, precisely aligning with the bacterial plant glycogen synthase subfamily. This gene is termed *SS1* and embodies two Pfam domains, Glyco_transf_5 and Glycos_transf_1, which typically confer the enzymatic function to elongate the glucose chain in glycosyl donors like UDP-glucose. The involvement of *SS1* in the glycosylation process is critical for the formation and storage of starch within plant tissues.

## 4. Discussion

The urgent conservation of the dioecious magnolia and its unique floral architecture has drawn attention within the botanical community regarding the underlying mechanisms governing its distinctive survival strategy. Our study aimed to elucidate the molecular intricacies directing leaf developmental processes in *M. kwangsiensis* and characterize transcriptional profiles to pinpoint pivotal genetic factors involved in the accrual of development-related bioactivate compounds and potential medicinal phytochemicals, especially the biosynthesis of bioactive polysaccharides and flavonoids. Through comparative transcriptomics of juvenile, tender, and mature leaf phases, we have unveiled intriguing insights into the dynamic genetic transitions that scaffold developmental milestones, confer specialized metabolic capacities, and prime the species’ ecological resilience.

Prominent among our findings is the substantial genomic reorganization underlying leaf developmental transitions, which is evidenced by thousands of differentially expressed genes cumulatively. Our results reveal parallels with previous studies defining three Magnoliaceae leaf development phases, each with unique genetic signatures: (1) an initial morphogenesis stage marked by cell division and developmental genes, (2) a leaf expansion phase featuring cell wall biosynthesis and cytoskeletal genes, and (3) a mature stage prioritizing photosynthesis and secondary metabolism [15,16,17]. The transcriptional shifts highlight significant gene regulatory changes across successive developmental stages. Detailed analysis shows the upregulation of critical processes aligning with phase-specific priorities. Trends in tender leaves mirror an accent on stress responses, potentially instilling resilience in nascent tissues. Mature leaves exhibit the conspicuous upregulation of polysaccharide biosynthesis and cell wall biogenesis genes, consolidating photosynthetic infrastructure and mechanical stability. Downregulation patterns demonstrate a withdrawal from cell proliferation as tissues transition toward specialized functionality. These molecular transitions neatly concur with the physiological progression from energetic investment in new biomass toward photosynthetic asset creation, agricultural yield, and chemical defense roles in mature leaves.

Nevertheless, particular deviations illuminate the preponderance of cell wall developmental genes in mature *M. kwangsiensis*, which contrasts with observations that photosynthesis and specialized metabolism attain primacy following Magnoliaceae leaf expansion. This disparity likely stems from indeterminate growth habits in woody perennials. Additionally, the *Magnolia* species’ subtropical evergreen nature and extensive secondary chemistry could impose selection pressures that necessitate continued investments in defensive compounds alongside photosynthetic activity through ontogeny. These facets may explain the simultaneous upregulation of “cell wall” and “secondary metabolism processes” in mature magnolia leaves.

Diving deeper into the maturation chronology of *M. kwangsiensis* leaves, we have cataloged over a hundred genes exhibiting concordant trajectories across our specimen series. This conserved signature set captures the molecular essence underpinning fundamental transitions in foliar development. Among these, histone variants signify chromatin reconfigurations that allow developmental genes to access target sites during tissue maturation [18,19]. Enriching histone genes points to global modifications in chromatin architecture eliciting precisely timed developmental events [20]. Moreover, proteins regulating DNA replication and repair highlight the proliferation of cells with actively dividing nuclei during morphological construction. The prominence of these fundamental activities underpins the expansion of cellular capacity necessary to establish photosynthetic real estate and scaffold demanding biosynthetic pathways operationalized later in development [21,22]. These preliminary investments prime tissues for downstream photosynthetic productivity, fueling plant growth and facilitating chemical defense roles [22,23,24].

The work substantially builds upon the biosynthesis of the chief bioactive constituents accelerated within mature leaves, which is concurred through the preponderance of cellular polysaccharide production genes discovered exclusively in this dataset. The combination of elevated cell wall investments and a surge in polysaccharide biosynthesis implies that specialized metabolism escalation complements structural enhancement in mature leaves. The mechanistic overlap between defensive chemistry and architectural robustness hints at intriguing complementary selection pressures that shape the duality of structure and function during leaf history [25,26,27]. Photosynthetic maturation thus proceeds with tissue resilience and chemical defense amplification to secure plant stability and survival [25,26,28].

Polysaccharide biosynthesis lies central to magnolia’s pharmaceutical utility [7]. Polysaccharides obtained from *M. kwangsiensis* have shown promising potential in contributing to human health. These complex carbohydrates can modulate the immune system, display anti-inflammatory actions, or act as antioxidants. Some polysaccharides are also known for their antitumor activities. The specific bioactive polysaccharides in *M. kwangsiensis* require in-depth characterization to fully understand their therapeutic value and the mechanisms behind their health benefits [29,30,31]. Hence, the induction of pertinent genes opens avenues for sustainable bioprospecting and conservation. Pathway analysis exposes the complexity of starch metabolism regulation with bifunctional enzymes like glycosyltransferases, starch synthases, and amylases dynamically expressed across development. These specialized proteins direct polysaccharide construction and degradation, manipulating chemical compositions to meet physiological needs. Aligned with magnolia’s evergreen longevity, the maintenance of such elaborate enzymatic machinery sustains year-round metabolic activity and a prolonged storage of bioactive metabolites synthesized over successive growth cycles. The lasting presence of this biochemically proficient tissue likely aids the species’ ability to perenniate in isolated subtropical localities through episodic flowering and temperate winters [7,8,16,17].

Our study’s integrated approach of GO and KEGG pathway analyses facilitated the discovery and characterization of four novel genes potentially central to the starch synthesis pathways. These identified genes—each displaying distinctive features and enzymatic functions relevant to polysaccharide biosynthesis—are postulated to have substantial roles in developing starch granules within the species’ leaf tissues. The noteworthy genes include Chr01.2399, annotated as a 1,4-α-glucan-branching enzyme, which is implicated in the generation of branch points in the polysaccharide chain during the creation of glycogen [32,33]; Chr02.481, identified as Isoamylase 3 (*ISA3*), exhibits homology to enzymes known for their capacity to cleave α-1,6 glycosidic bonds in amylopectin [32,34]; Chr02.4751 is associated with the ADP-glucose synthesis pathway and has been denoted as encoding a protein that catalyzes the synthesis of the activated glycosyl donor, ADP-glucose from Glc-1-P and ATP [35,36]; and Chr04.195, which is part of the glycosyltransferase 1 family under the bacterial plant glycogen synthase subfamily [37,38]. The discovery of these genes aligns with previous research from other trees that glycosyltransferase is critical to plant development, enriching our current understanding of the exact member of genes that regulate the leaf tissue’s unique metabolic demands in *M. kwangsiensis*.

Comparing our Magnolia transcriptome results to Arabidopsis ortholog expression provides evolutionary insight into conserved gene regulation cascades governing leaf maturation among flowering plants. Our transcriptomic investigation also hints that some findings within *M. kwangsiensis* could broaden our understanding of other Magnolia members and potentially additional plant species. Our data reveal 653 to 2919 differentially expressed genes per developmental comparison including numerous Arabidopsis orthologs, suggesting conserved underlying genetic programs directing the emergence of sophisticated leaf functionality transcending the diverse morphologies of magnoliid and core eudicot clades. As ancestral angiosperms, some Magnolia organogenesis genes may be conserved across plants. Despite over 140 million years divergence, over 60% Magnolia transcriptome orthology points to ancestral developmental pathways retained since magnoliids and eudicots shared a common ancestor [39,40,41].

In summary, our research provides a fundamental understanding of how genes shape leaf growth in an endangered Asian tree highly regarded for its medicinal attributes. We have identified critical genetic shifts that guide leaf development from one stage to the next. This includes activating genes that strengthen leaf structure, ensuring durability and effective photosynthesis alongside a suite of enzymes that generate medicinal compounds. The coordination between these processes appears finely tuned to the plant’s environmental demands. Furthermore, our study reveals a potential genetic link connecting the production of Magnolia’s primary polysaccharides to cell wall formation. This discovery suggests a strategic amalgamation of physical strength and chemical defense within the plant—a concept that could redefine our perspective on the convergent evolution of such survival strategies in ancient vascular plants. Our work provides a detailed view of the genetic mechanisms by which *M. kwangsiensis* balances structural integrity with the synthesis of defensive chemicals. This insight is precious for conserving unique plant species in Asia, paving the way for applying biotechnological approaches in plant science. The ultimate goal is to harness the full potential of specialized plant metabolism sustainably, aligning biotechnological innovation with the preservation of natural ecosystems.

## 5. Conclusions

This study conducted a comparative transcriptome analysis across juvenile buds, tender leaves, and mature leaves of the Dioecious Magnolia *M. kwangsiensis*, highlighting expressional and genetic conserved and divergent traits during developmental stages. The comprehensive transcriptomic overview and the discovery of core sets of differentially expressed genes provide a foundational understanding of the species’ genetic makeup, mainly reflecting on pathways essential for survival and pharmaceutical exploitation. GO and KEGG enrichment outlined genes associated with carbohydrate and polysaccharide biosynthetic processes in mature leaves, which is particularly noteworthy. Four new genes encoding glycosyltransferases were identified. This study underscores the importance of conserving the Dioecious Magnolia while paving the way for future biotechnological applications of its valuable metabolites.

## Figures and Tables

**Table 1:** Overview of the size, quality, and mapping rate of transcriptome sequencing data.

Groups	ID	Num_Seqs	Sum_Len (bp)	FR_Q30 (%)	FR_GC (%)	RR_Q30 (%)	RR_GC (%)	Mapping Rate (%)
mature leaves	KL2	21,108,115	3,166,217,250	93.46	47.3	89.92	47.33	93.53
mature leaves	KL4	20,800,796	3,120,119,400	93.45	47.34	90.09	47.37	93.45
mature leaves	KL5	20,735,915	3,110,387,250	93.6	47.35	89.28	47.39	93.68
buds	KL6	19,053,880	2,858,082,000	93.29	46.29	90.44	46.33	93.26
buds	KL7	19,163,063	2,874,459,450	93.35	46.37	90.14	46.4	93.53
buds	KL8	20,774,753	3,116,212,950	93.43	46.78	91.04	46.83	93.73
tender leaves	KL9	20,996,356	3,149,453,400	93.35	46.25	91.69	46.31	91.76
tender leaves	KL11	21,645,506	3,246,825,900	93.4	46.59	90.99	46.62	92.85
tender leaves	KL12	22,872,339	3,430,850,850	93.14	46.09	89.23	46.12	93.21

Notes: Num_seqs and sum_len are the shorts for the number of sequences and summed length of sequencing data, respectively. FR and RR are the abbreviations of forward and reversed reads, respectively. Mapping rates are collected from hisat2 log files.

**Table 2:** Functional descriptions of the 4 genes enriched by the GO term ‘polysaccharide biosynthetic process’ and the KEGG pathway of ‘starch and sucrose metabolism’.

ID	Egg-NOG Des	Gene Name	Pfam Domain
Chr01.2399	1,4-α-glucan-branching enzyme	-	α-amylase, α-amylase_C, CBM_48
Chr02.481	Isoamylase 3	*ISA3*	α-amylase, CBM_48
Chr02.4751	This protein plays a role in the synthesis of starch. It catalyzes the synthesis of the activated glycosyl donor, ADP-glucose from Glc-1-P and ATP.	*ADG2A*	NTP_transferase
Chr04.195	Belongs to the glycosyltransferase 1 family. Bacterial plant glycogen synthase subfamily	*SS1*	Glyco_transf_5,Glycos_transf_1

Note: Gene IDs are inherited from reference genome annotations with the prefix ‘Chr’ followed by numbers indicating the corresponding chromosome location. Functional descriptions are obtained through eggNOG-mapper annotations, which provide an evolutionary genealogy of gene functions.

## Data Availability

Raw RNA-seq sequencing data were uploaded to the NCBI SRA database under the BioProject ID of PRJNA1057110 and BioSample accession of SAMN39120375.

## Supplementary Materials

The following supporting information can be downloaded at https://www.mdpi.com/article/10.3390/genes15030335/s1, Supplement Figure S1: Two ecological photos for the buds, tender and mature leaves of *M. kwangsiensis*. Supplement Table S1: Calculated FPKM values for all nine sequencing samples. Supplement Table S2: Egg-NOG mapper functional annotations of the 124 DEGs among all three comparison groups. Supplement Table S3: GO enrichment analyses of the DEGs among all three comparison groups. Supplement Table S4: KEGG enrichment analyses of the DEGs among all three comparison groups.
