# Supplementary figures and images for: Transcriptomics of Leaf Development in the Endangered Dioecious Magnolia kwangsiensis: Molecular Basis Underpinning Specialized Metabolism Genes
Source: Genes (Basel). 2024 Mar 4;15(3):335. doi: 10.3390/genes15030335 (PMC10970092; doi:10.3390/genes15030335)

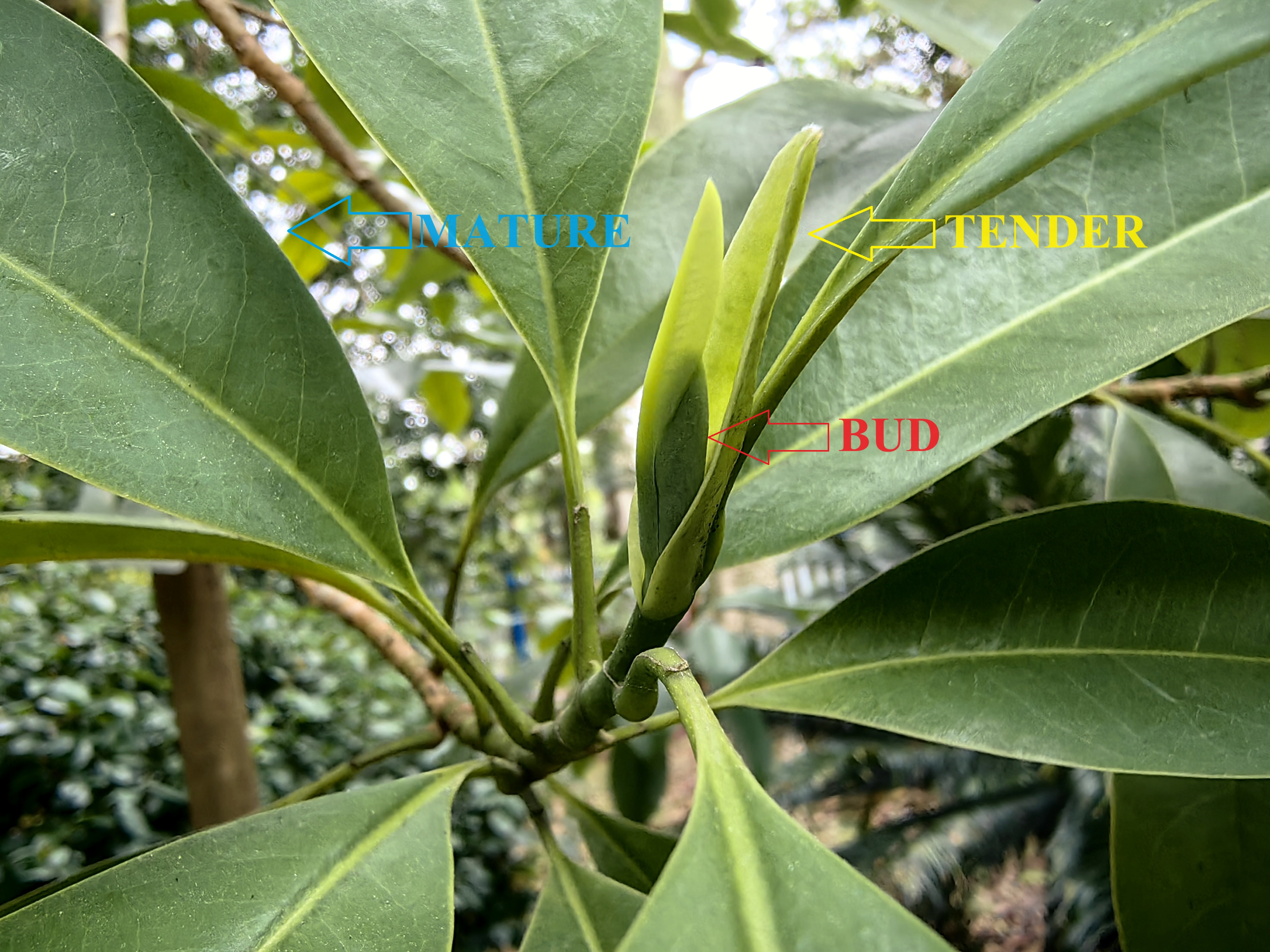

Supplement: Supplementary file 1 [file genes-15-00335-s001.zip › FigS1_A.jpg]

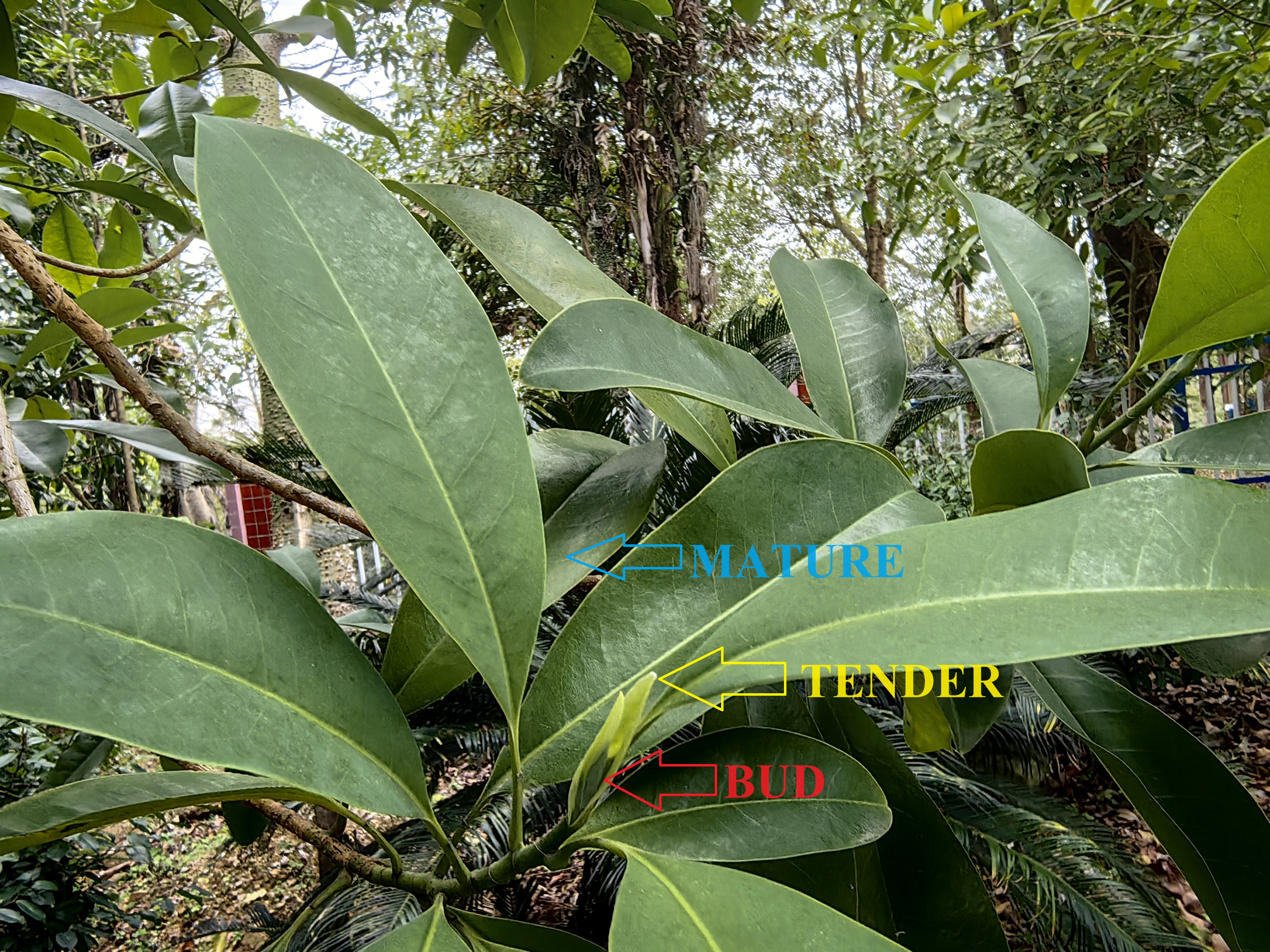

Supplement: Supplementary file 1 [file genes-15-00335-s001.zip › FigS1_B.jpg]
